# Pulmonary Artery Thrombosis: A Diagnosis That Strives for Its Independence

**DOI:** 10.3390/ijms21145086

**Published:** 2020-07-18

**Authors:** Olga Porembskaya, Yana Toropova, Vladimir Tomson, Kirill Lobastov, Leonid Laberko, Viacheslav Kravchuk, Sergey Saiganov, Alexander Brill

**Affiliations:** 1 Mechnikov North-Western State Medical University, Saint Petersburg 191015, Russia; VyacheslavKravchuk@szgmu.ru (V.K.); sergey.sayganov@szgmu.ru (S.S.); 2 Institute of Experimental Medicine, Saint Petersburg 197376, Russia; 3 Institute of Experimental Medicine, Almazov National Medical Research Center, Saint Petersburg 197341, Russia; yana.toropova@mail.ru; 4 Pavlov University, Saint Petersburg 197022, Russia; nic.spb@mail.ru; 5 Pirogov Russian National Research Medical University, Moscow 117997, Russia; lobastov_kv@hotmail.com (K.L.); laberko@list.ru (L.L.); 6 Institute of Cardiovascular Sciences, College of Medical and Dental Sciences, University of Birmingham, Birmingham B152TT, UK; 7 Department of Pathophysiology, Sechenov First Moscow State Medical University (Sechenov University), Moscow 119991, Russia

**Keywords:** pulmonary artery thrombosis, pulmonary embolism, prothrombotic phenotype, platelet activation

## Abstract

According to a widespread theory, thrombotic masses are not formed in the pulmonary artery (PA) but result from migration of blood clots from the venous system. This concept has prevailed in clinical practice for more than a century. However, a new technologic era has brought forth more diagnostic possibilities, and it has been shown that thrombotic masses in the PA could, in many cases, be found without any obvious source of emboli. Chronic obstructive pulmonary disease, asthma, sickle cell anemia, emergency and elective surgery, viral pneumonia, and other conditions could be complicated by PA thrombosis development without concomitant deep vein thrombosis (DVT). Different pathologies have different causes for local PA thrombotic process. As evidenced by experimental results and clinical observations, endothelial and platelet activation are the crucial mechanisms of this process. Endothelial dysfunction can impair antithrombotic function of the arterial wall through downregulation of endothelial nitric oxide synthase (eNOS) or via stimulation of adhesion receptor expression. Hypoxia, proinflammatory cytokines, or genetic mutations may underlie the procoagulant phenotype of the PA endothelium. Both endotheliocytes and platelets could be activated by protease mediated receptor (PAR)- and receptors for advanced glycation end (RAGE)-dependent mechanisms. Hypoxia, in particular induced by high altitudes, could play a role in thrombotic complications as a trigger of platelet activity. In this review, we discuss potential mechanisms of PA thrombosis in situ.

## 1. Introduction

In the middle of the 19th century, Rudolf Virchow, studying pathophysiological aspects of venous thromboembolism (VTE), put forward a theory that remained unshakable for a century and a half. Virchow argued that thrombotic masses are not formed in the pulmonary artery (PA) but result from migration of blood clots from the peripheral venous system, causing secondary obstructions in the branches of the PA [1]. 

Since then, there have been attempts to match the morphological characteristics of thromboemboli with possible sources of their formation [2]. Since emboli were considered to be thrombotic casts from the veins, it was believed that blood clots with a large diameter originated from large veins, whereas smaller ones were from the crural or pelvic veins. If no thrombi could be discovered in the deep veins, transformation of a thrombus into an embolus by emptying of a vein was believed to be the cause of embolism [2].

This mechanistic approach is still dominant in clinical practice. However, concurrent with the accumulation of clinical data on VTE complications, the idea of embolism from thrombotic masses in the venous system as the only source of emboli in the branches of the PA started to become doubtful, since no initial thrombus was found in a large number of patients [3,4]. Cases of thrombi discovered in the branches of the PA in the absence of concomitant deep vein thrombosis (DVT) cannot be justified only by the drawbacks of evaluation techniques that are used to diagnose thrombosis. Magnetic resonance (MR) panphlebography assessment of the venous system of the whole body confirms the possibility of thrombosis in the PA with no thrombi in the deep veins. In a Dutch study of 102 patients with newly discovered symptoms of pulmonary embolism (PE), DVT was diagnosed by panphlebography in 44% of patients [5]. No primary source of PE was identified in 55 patients (56%) and the reliability of the results obtained in the study were confirmed by the high sensitivity (96%) and specificity (98%) of MR phlebography in the diagnostics of venous thrombosis [5,6].

## 2. Clinical Aspects of Pulmonary Thrombosis

**Figure 1:**
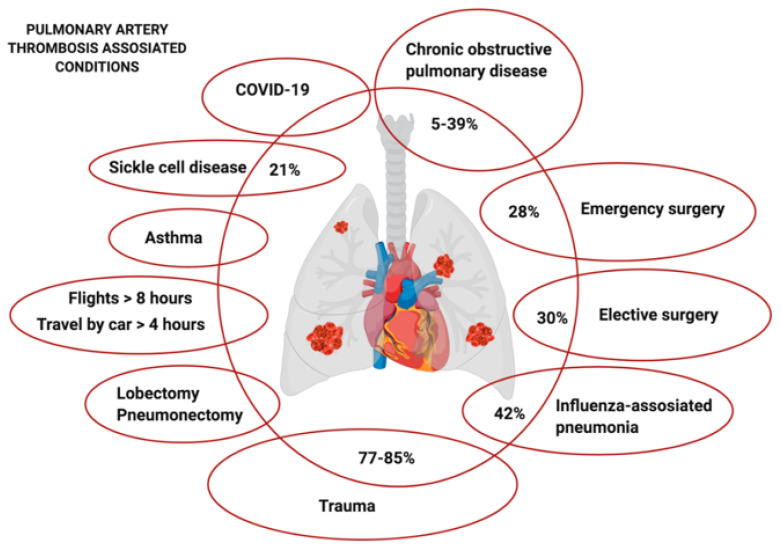
Diseases and conditions with high risk of pulmonary artery thrombosis. Percentages show patients with clots in the lungs and without clots in the deep veins.

Development of thrombosis in the branches of the PA has been reported in pulmonological, hematological, surgical, and infectious patients (Figure 1). Incidence of thrombotic obstruction of PA branches in the Registro Informatizado de la Enfermedad TromboEmbolica venosa (RIETE) study, which involved 2984 patients with chronic obstructive pulmonary disease (COPD), was 59% [7]. These included both patients with true PE and with isolated involvement of the PA and no DVT. However, follow-up of these patients for 7 days and 3 months showed that rethrombosis in the territory of the PA (without DVT), which predominates in this type of patients, is of greater importance in the structure of recurrent VTE and mortality [7,8]. Analysis of exacerbations of COPD showed that low SpO_2_ values, dyspnea, and disease severity increase the risk of PA thrombosis [9]. Studies evaluating incidences of local PA thrombosis separately from DVT+PE reported its development in 5–39% of patients with COPD [10,11]. Computed tomographic (CT) angiography findings provide evidence of the most common involvement of segmental (4.7%) and subsegmental (18.6%) PA branches [12]. Thrombi in the PA trunk are diagnosed in 5.8% of cases only [12]. However, data on the incidence of PA branches involvement in patients with COPD vary across different studies [10].

A high risk of PA thrombotic complications is also typical for patients with asthma [13]. The risk of PA thrombosis is significantly increased in patients with severe and moderate asthma, compared to the general population [13]. At the same time, there is no association with the high risk of DVT in such patients [13].

A high risk of pulmonary thrombosis is associated with episodes of aggravation in sickle cell anemia (SCA) [14]. A complication presented as acute thoracic syndrome (ATS) during the period of aggravation in SCA is caused by erythrocyte hemolysis, vasoconstriction, and platelet aggregation and may be accompanied by thrombosis of the PA branches. Computed tomographic (CT) imaging performed within the first 3 days of ATS identifies pulmonary thrombosis without DVT in 21% of patients [14]. Thrombi are discovered mostly in the segmental branches of the PA (75%) and less commonly in its subsegmental branches (15%) [14]. Thrombi are also found in the lobar branches of the PA, although in a lesser number of cases (10%) [14]. A typical CT finding is the opartial artery filling defect. Thrombotic complications are registered both in patients with the newly developed ATS and those with recurrent ATS and in some cases during each exacerbation [14]. 

A high incidence of pulmonary thrombotic events (reaching 9.3%) is one of the complications of gunshot wounds and their associated injuries [15]. In this cohort of patients, the combination of PA thrombosis with DVT was registered only in 15–23% and primary PA thrombosis in more than 75% [3,15]. PA thrombosis is diagnosed in the first 2 h following the moment of injury. The causes of “acute peritraumatic pulmonary thrombosis” may differ from those in other diseases and may be due to the local response of the PA to injury [15]. Formation of atelectasis, vascular constriction, blood stasis, and local hypoxia may be significant as well [16]. The distribution of thrombi in “acute peritraumatic thrombosis” of the PA is registered in the segmental (38%) and subsegmental (15%) branches of the PA [15]. Thrombi can also be detected with high incidence in the large branches of the PA (47%) [15].

It has been shown that pulmonary thrombosis can occur in the long-term postsurgical period of patients that have undergone a pneumonectomy or lobectomy [17]. The average period of time between the surgical intervention and development of this complication is 97 (37–231) days. Assessment of the degree of recanalization of thrombosed branches of the PA showed better results in patients with PE than in those with thrombosis of the PA. The degree of recanalization was 90% and 25%, respectively. 

Based on the results of postmortem autopsy, the source of PE could not be detected in 28% of patients who died after emergency surgery and in 30% of patients after elective surgery [4]. Among patients who died in the non-surgical departments, thrombosis of the PA without DVT was found in 20.9% [4]. The data were obtained from the findings of autopsies of 500 patients with PE being a confirmed cause of death. After excluding all cases where the source of embolism was identified, the cases were designated as PE with no primary source [4].

Thrombosis of the PA can develop as a complication in viral infections. In the Multi Environmental and Genetic Assessment (MEGA), with a case-control population study of 2069 patients with VTE (PE +/− DVT), a high risk of pulmonary thrombosis was demonstrated in patients who had experienced viral pneumonia within a year prior to this event [18]. At the same time, the risk of primary thrombosis of the PA increased 8.1-fold (95%; 6.2–10.6), significantly exceeding that for DVT (3.0; 95%, 2.2–4.0). Thrombosis of the PA without DVT could also be observed in patients with complicated severe viral pneumonia secondary to influenza [19,20]. 

Studies of viral pneumonias caused by severe acute respiratory syndrome coronavirus 2 (SARS-CoV-2) indicate that the development of pulmonary thrombosis is frequent and typical for this disease [19,21,22]. A comparison of the incidence of PA thrombosis in severe pneumonias caused by SARS-CoV-2 and by the influenza virus demonstrates the higher risk for this complication in patients with COVID-19 (22.88% versus 14.4%, respectively) [19]. The combination of PA thrombosis with DVT is registered in only 13.6% of patients with COVID-19, which suggests predominantly primary PA thrombosis. As a rule, bilateral thrombosis of the PA (40%) is described with predominant localization of thrombotic masses in the segmental branches of the PA (55%) [19].

During the 2003 epidemic of viral pneumonias caused by the SARS virus of the coronavirus family, there were also reports of venous thrombotic complications in patients with this disease [23,24]. In some patients, pulmonary thrombosis was primary and not associated with DVT [24]. CT angiography showed localization of thrombi in the large branches of the PA [23]. 

Cases of cytomegalovirus infection accompanied by PA thrombosis have been described in patients without immunodeficiency [25]. Thrombosis is preceded by a vivid clinical presentation in the form of fever persisting for several days, lymphadenitis, and asthenia; diarrhea may also be observed [25,26]. Giant basophilic lymphocytes are detected in the blood, thrombocytopenia is possible, and high C-reactive protein (CRP) values are registered; an increase in hepatic transaminases levels is found in some patients [25,26]. Development of sudden dyspnea, chest pain, and a nonproductive cough may be signs of primary pulmonary thrombosis in these patients [26]. 

The MEGA population study evaluated specific features of the course of a first episode of VTE, depending on the presence of either Factor V Leiden or the prothrombin gene 20210A mutation [27]. Over the 5 years of the study, PA thrombosis was diagnosed in 885 patients, and DVT and DVT+PE in 2063 and 365 patients, respectively. The presence of Factor V Leiden factor mutation increased the risk of isolated DVT 8-fold (Odds ratio, OR 7.7 95%; 3.9–15.3) and the risk of isolated thrombosis of the PA only 1.5-fold (OR, 1.6 95%; 0.7–2.7) compared to the patients without this mutation. This feature was called the Leiden paradox. The discovered differences did not affect either the density of the thrombus or the rate of its formation in patients from the two groups. The presence of the prothrombin gene mutation equally increased both risks for development of DVT (OR 3.2 95%; 2.4–4.2) and PA thrombosis (OR 2.3 95%; 1.5–3.3) [27]. 

The prolonged stay of a person at a high altitude above sea level is known to increase his/her risk of thrombotic events. The risk for development of VTE in persons residing for 11 months at an altitude of more than 3000 m above sea level increases 30-fold [28]. Unfortunately, there is no separate analysis of the groups of patients with PE and pulmonary thrombosis in the study. However, reviewing again the individual clinical cases could give the information about the isolated PA thrombosis with involvement of its large and segmental branches in patients from a high altitude [29].

Increased homocysteine content can become a provoking factor for thrombosis in large branches of the PA, with development of a vivid clinical presentation resembling PE symptoms: dyspnoe, productive cough with hemoptysis, and right sided pleuritic chest pain [30,31]. Similar events may occur in cases where there is a combination of high homocysteine content with pernicious anemia and hereditary thrombophilia [32].

Flights lasting more than 8 h and 4-h and longer driving trips can also provoke not only DVT but also primary PA thrombosis [5].

## 3. Potential Mechanisms for Primary Thrombosis in the PA

### 3.1. Structural Features and PA Endothelium Molecules Potentially Relevant to Thrombosis

Pulmonary arterial endothelium is a complex, metabolically active system that fulfills a barrier function and is involved in gas exchange, the regulation of vascular tone, the coagulation system, leukocyte diapedesis, and vascular permeability [33]. PA endothelium forms a monolayer due to intercellular interaction proteins, structural cellular proteins, and cytoskeletal proteins [33]. Among the structural proteins of endothelial cells, an important role is played by caveolin-1 that mediates regulation of nitric oxide (NO) production and protein C effects on protease mediated receptor 1 (PAR-1) [34]. The properties of caveolin can be changed as a result of external causes, including oxidative stress [35]. Suppression of caveolin-1 causes endothelial dysfunction and becomes one of the mechanisms for the development of pulmonary hypertension, which carries the risk for PA thrombosis [36,37,38,39].

Endothelial nitric oxide synthase (eNOS) directly binds to caveolin-1 [40]. This protein negatively regulates eNOS function and NO production [40]. The prominent role of NO in antithrombotic mechanisms is the prevention of platelet adhesion to the vessel wall [41]. It has been shown that infusion of NO inhibitors increases platelet deposition in the vessels [41].

Protein C activation occurs by its interaction with thrombin coupled with thrombomodulin. Activated protein C (APC) binds its own endothelial protein C receptor (EPCR), which is associated with caveolin-1 on the membranes of endotheliocytes. The interaction with APC provides dissociation of EPCR from the caveolin-1 and further EPCR binding to PAR-1, which blocks the effects of thrombin on vascular permeability [34]. The EPCR receptor is scattered throughout the PA endothelium from the large branches to the minor capillaries [42]. Mutant inactive protein C infusion deprives the complex with EPCR of its protective function, but in an experimental setting, does not prevent the lethal effect of injected thrombin, since the death of mice still results from PA thrombosis [43,44]. Suppression of protein C may result from the impact of tumor necrosis factor (TNFα) and reactive oxygen species (ROS) [45,46].

The endothelial permeability regulation is only one of many different functions of PAR, which itself presents a molecular link between coagulation factors and immune cells involved in thrombosis [47]. The PAR receptor family has four members. PAR-1,3,4 are activated by thrombin and PAR-1 and 2 by the TF-VIIa and Xa complex [48]. Thrombin binding to PAR receptors on the PA endothelium causes a number of effects: formation of thrombi, increase of vascular permeability, secretion of pro-inflammatory cytokines, chemoattractants and adhesion molecules, and upregulation of platelet aggregation and procollagen production [45,47]. 

The role of the thrombin-PAR-1 interaction in thrombosis was demonstrated by real-time tracking of probes labelled with fluorophores involved in this protease-activated thrombotic process [49]. Intravenous administration of non-lethal doses of thromboplastin accompanied with picomolar amounts of labelled probes to mice increased clotting activity in the PA. Administration of a thrombin inhibitor, hirudin, reduced thrombus formation in the PA.

An important factor that mediates homeostasis in the PA is the glycocalyx layer on the endotheliocytes. Destruction of glycocalyx results in the loss of heparan sulfate and exposure on the surface of endothelial cells of adhesion molecules inter-cellular adhesion molecule (ICAM-1) and vascular cell adhesion molecule (VCAM-1), which become accessible for circulating leukocytes involved in thrombosis [50,51]. One of the components of glycocalyx is the proteoglycan endocan [52]. Thus, expression of endocan is specific to the endothelium of the pulmonary and renal arteries [52]. Increase in blood endocan levels in patients with CT angiographic evidence of a pulmonary thrombotic event is associated with a more severe course of the disease [52]. Blood endocan levels rise with increased PA pressure and decreased partial oxygen tension.

PA endothelial glycocalyx transmits mechanical signals from the endothelial surface to the cytoskeleton in response to varying shear stress and causes vessel contraction due to the interaction of actin with myosin [53,54]. Increased contractions in the case of PA pressure fluctuations result in dissociation of cadherin membrane proteins, extension in intercellular spaces, and enhanced expression of adhesion molecules, leading to the recruitment of neutrophils and monocytes, which contribute to thrombosis [53,54].

The procoagulant shift of the PA endothelium can be mediated by a number of factors, such as vWf, P-selectin, TNF, ROS, and modulation of the activity of eNOS and APC PKC and the function of glycocalyx.

### 3.2. The Role of TNFα in Activation of PA Endothelium

TNFα has been shown to activate PA endothelium in vitro [55]. In human cultured PA endothelial cells, TNFα induces the procoagulant phenotype within 24 h. This occurs in particular via increased expression of ICAM-1, decreased expression of EPCR, and suppressed thrombomodulin secretion. In addition, expression of tissue plasminogen activator (tPA) and urokinase-type (uPA) is observed, accompanied by PAI-1 upregulation. 

In clinical practice, there is a number of conditions accompanied by the production of pro-inflammatory cytokines, such as TNFα, including the postsurgical period, ulcerative colitis, injuries, sepsis, and others [56,57,58].

### 3.3. The Role of Hypoxia in the Activation of PA Endothelium

Endothelial activation can be induced by hypoxia, which provokes secretion of the ROS and production of hypoxia inducible factors (HIFs) 1 and 2 in the endothelial cells [59,60]. With a decrease in oxygen concentration, HIFs accumulate in the cell nucleus and bind to the hypoxia-responsive element (HRE) on the promoter or enhancer of targeted genes, which activates their transcription [61,62]. HIFs induce synthesis of pro-inflammatory factors and adhesion receptors, including ICAM-1, nuclear factor-kappaB (NF-kB), TNFα, and Interleukin-6 (IL-6), as well as tissue factor (TF) and PAI-1, which promote blood coagulation [62]. Protein S and tissue factor pathway inhibitor (TFPI) expression is inhibited by HIFs [62]. In the setting of hypoxia, other signaling pathways are also activated, such as early growth response-1 (EGR-1), which activate the PAI-1 genes and thus contribute to the development of thrombosis [63]. Expression of hypoxia-induced genes is increased in persons residing at more than 3000 m above sea level and who have had DVT in the past [64].

Introduction of microspheres into the PA in an experiment showed that secretion of HIF1α and HIF2α occurs in response to hypoxia not only locally in the areas with occlusion of the blood vessel lumen, but also in adjacent segments where there is no obstruction [65]. 

In patients with a prolonged history of smoking, combined with chronic bronchitis, there is a significant increase of HIF1α and vascular endothelial growth factor (VEGF) in the vascular endothelium and smooth muscle cells (SMCs), compared to the smokers without chronic bronchitis [66,67]. Similar changes have been observed in patients with bronchial asthma [68]. A different presentation is typical in patients with COPD in whom the mechanisms of cell degradation prevail over the mechanisms induced by hypoxia. HIF1α and VEGF levels in these patients are significantly reduced compared to those in smokers with no COPD. This is combined with severe apoptosis of endothelial cells and vascular atrophy [69,70]. An increase in the activity of enzymes, including serpin family F member 1 (SERPINF1, protease inhibitor), also results in the suppression of angiogenesis and the apoptosis of endotheliocytes [66,71]. Endothelial atrophy causes loss of anti-adhesive and antithrombotic functions of the endothelium [66].

ROS enhance the risk of thrombus formation through several mechanisms. They increase expression of TF on endothelial cells and monocytes, inactivate protein C and its agonist thrombomodulin, and promote oxidation of fibrinogen, which increases its conversion to fibrin [46]. Free oxygen radicals in mitochondria in the setting of hypoxia activate PAR-1 and PAR-2 receptors, leading to TF secretion [72]. In the context of hypoxia, PAR-1 activates the nuclear transcription factor NF-kB, which regulates secretion of pro-inflammatory cytokines and expression of neutrophil ligands on other cells [33,72,73]. Hydrogen peroxide produced in the endothelial mitochondria of pulmonary micro-vessels is involved in the upregulation of the pro-inflammatory response [74]. Increased pressure in the pulmonary capillaries contributes to increased production of mitochondrial ROS, primarily hydrogen peroxide, provoking exocytosis of the Weibel–Palade body constituents, in particular P-selectin [74].

### 3.4. The Role of Genetic Factors in the Functioning of PA Endothelium

Genetic changes may underlie the procoagulant phenotype of the PA endothelium. Hereditary factors play a role in the development of pulmonary hypertension, including a heterozygous mutation in the gene Bone Morphogenetic Protein Receptor Type 2 (BMPR2), found in 50–70% of patients with familial pulmonary hypertension [39]. The same mutation has been identified in 11–40% of patients with idiopathic pulmonary hypertension. BMPR2 is expressed in a number of cell types; however, this heterozygous mutation is of the greatest importance, particularly in the PA endothelium, because of its role in pulmonary hypertension. In experiments with BMPR2 knockout mice, minor branches of the PA are partially or completely occluded by fibrin(ogen) positive thrombi in 53% of cases [39]. This likely results from damage of the endothelial cells and apoptosis by BMPR2 signaling disregulation, which contributes to inflammation and thrombosis in pulmonary hypertension [39].

### 3.5. Potential Role of Viruses in Predisposition to PA Thrombosis

Endothelial damage can be caused by the immediate effect of a virus that destroys the cell. The ability of the influenza virus to penetrate cells has been shown in vitro in monolayers of endothelial cells [75]. After 3 h incubation, expression of TF was observed, which peaked at 24 h [75]. Destruction of the endothelial cells was detected 36–48 h later.

Histological evaluation of the material collected from patients infected with SARS-CoV-2 demonstrates immediate damage to the endothelial cells from various vascular beds [20,76]. Viral particles were found in the endothelium of the lungs, kidneys, and small bowel. Signs of endotheliitis and endothelial edema were observed, and apoptotic bodies, lymphocytic and leukocytic infiltration, and platelets were discovered [76,77,78]. Apoptosis and pyroptosis may play a significant role in the death of endotheliocytes and the development of endotheliitis in COVID-19 [76]. The mechanism of penetration of coronavirus into the cell is based on its interaction with angiotensin converting enzyme II (ACEII) receptors [79,80]. Expression of ACE-II is typical for PA endotheliocytes, making them a target for SARS-CoV-2 [81]. This may explain the high prevalence of PA thrombosis in COVID-19 infection and targeting ACE-II could potentially be beneficial to tackle this dangerous complication.

## 4. The Role of Platelets in PA Thrombosis

Activated platelets likely play a role in PA thrombosis. It has been shown in the Warfarin and Aspirin (WARFASA) and Aspirin to Prevent Recurrent Venous Thromboembolism (ASPIRE) trails that the inhibition of platelet activity by antiplatelet drugs reduces the risk of recurrent PA thrombotic events after anticoagulant treatment is discontinued [82,83]. Inhibition of platelet activation by a coumarin-derived compound, Auraptene, or a Mer tyrosine kinase (MERTK) inhibitor, UNC2025, protects animals from death caused by PA thrombosis in experiments [84,85].

There are several potential mechanisms of platelet activation possible in PA thrombosis, which are discussed below.

### 4.1. PAR-Dependent Platelet Activation

In contrast to the endotheliocytes, only two types of PAR receptors are expressed on human platelets: PAR-1 and PAR-4 [86,87,88,89]. PAR-1 has a greater affinity for thrombin and therefore requires lower thrombin concentrations for activation [87]. Engagement of PAR-1 induces rapid platelet activation in a Ca^2+^-dependent fashion [87].

Activated platelets induce expression of adhesion molecules, such as ICAM-1, VCAM-1, vWf, and TF on the endothelial surface, thereby contributing to the migration of leukocytes, platelet adhesion to the endothelium, and blood clotting [51,90,91]. Binding of P-selectin on activated platelets to the P-selectin glycoprotein ligand (PSGL-1) on leukocytes causes the formation of leukocyte-platelet complexes and results in leukocyte activation [92].

The role of PAR receptor-mediated platelet activation in PA thrombosis has been shown in experiments with mice deficient in PAR-3 (an analogue of PAR-1 in humans) and PAR-4. Administration of thromboplastin into wild type Par3^+/+^ and Par4^+/+^ mice causes quick death of most of the animals because of the occlusion of the PA branches [86]. A similar mortality level is observed in Par3^+/-^ and Par4^+/-^ mice. In contrast, most Par3 ^−/−^ and Par4^−/−^ mice survived. Pulmonary artery perfusion and histological evaluation demonstrated much higher thrombosis prevalence in wild-type mice than in Par3 ^−/−^ mice [86].

### 4.2. HMGB1-Dependent Platelet Activation

A number of conditions are accompanied by the activation of platelets and leukocytes through the high-mobility group box 1 (HMGB1), a protein bound to DNA and released from the nucleus of dying or damaged cells, causing inflammation [93]. HMGB1 binding to receptors for advanced glycation end (RAGE) products and toll-like receptor (TLR4) induces platelet activation and granule secretion [93]. Platelet activation can be induced not only by exogenous HMGB1 but also by secretion of intrinsic HMGB1 [94]. This type of autocrine regulation promotes platelet aggregation during thrombosis formation [95]. The oxidized form of HMGB1 activates leukocytes and induces secretion of pro-inflammatory cytokines [96]. Platelet-derived HMGB1 contributes to the recruitment of another important participant in thrombosis, monocytes, enhancing their production of TF [95]. HMGB1 is present in PA thrombi and is also involved in the formation of neutrophil extracellular traps (NETs), which may contribute to PA thrombosis [97]. 

Platelet activation by HMGB1 may be observed in cases of injury, sepsis, myocardial infarction, hemorrhagic shock, and DVT [93]. On the first and second days after an injury, HMGB1 on patient platelets is increased [93]. In an experiment with mice under conditions of multiple trauma and hemorrhagic shock, HMGB1-dependent platelet aggregation occurs within the first 30 min. This is accompanied by platelet sequestration to the arteries of the lungs and formation of thrombi in the minor branches of the PA, as evidenced by histology [93]. In addition to the activation of platelets, this may be caused by the activation of the PA endothelium, which results from high concentrations of cytokines typical to multiple trauma [98]. Vasoconstriction of the PA branches, hypoxia due to constriction of the smaller PA branches, and shunting blood flow as a result of hemorrhagic shock aggravate activation of the PA endothelium, enhancing thrombosis [99].

### 4.3. The Role of Hypoxia in Platelet Activation

Hypoxia causes platelet hyperreactivity [100]. Platelets from acute COPD patients express higher levels of HIF2a than platelets from healthy people [60]. In the patients with blood PaO_2_ below 60 mmHg, the levels of HIF2α and PAI-1 in platelets are augmented [60]. Hypoxia induces shedding of extracellular vesicles from platelets, which is accompanied by increased expression of both HIF2α and PAI-1. Moreover, platelets from the patients are primed to produce HIF2a in response to either hypoxia or activation with thrombin [60]. 

In experiments on rats, high altitude hypoxia promoted platelet adhesion to collagen and fibrinogen [100]. Increases in secretion of dense granules reflects greater platelet activation [100].

High values of soluble P-selectin remain in the blood plasma of patients after VTE, residing at more than 3000 m above the sea level, and of patients with atherothrombotic vascular conditions, which indicates constant platelet activation [100,101]. Enhanced levels of soluble CD40L, P-selectin, and platelet factor-4 (PF-4) reflect platelet reactivity in high altitude hypoxia and could play a significant role in thrombotic complications [102]. 

### 4.4. Immune Response and Platelet Activation

Involvement of platelets in primary PA thrombosis may be due to their antiviral activity and involvement in the immune response. Megakaryocytes with significant hyperchromasia and an atypia of the nucleus associated with activated platelets have been described among the pathological findings in patients infected with SARS-CoV-2, who died of thrombosis in the small branches of the PA [77]. These were located in the minor blood vessels and capillaries of the lungs.

Megakaryocytes in the lungs are a source of about 50% of platelets in the body [103]. The encounter of megakaryocytes and platelets with an infectious agent results in the activation and initiation of the immune response. The signaling pathway, involving megakaryocytes and platelets in the immune response, may differ depending on the causative agent of the disease and may be mediated by the effects on interferon-induced transmembrane protein 3 (IFITM3) receptors or through pattern recognition receptors (PRR), which include different classes of TLR, C-type lectin receptor (CLR), and nucleotide-binding and oligomerization domain (NOD)-like receptors (NLRs) [104,105,106]. As a result, thrombotic function of platelets is upregulated through several mechanisms, such as stimulation of the TLR-3 receptor, the TLR-9-MyD88 signaling pathway, or FcγRII [107,108,109,110]. The result is secretion of the granule constituents, adhesion of the platelets and leukocytes to the endothelium, and release of NETs, which, combined, cause an alveolar-capillary barrier disruption in the lungs and may lead to thrombosis in the PA branches [111,112,113].

### 4.5. RAGE-Dependent Activation of Endotheliocytes and Platelets and Release of NETs

Activation of PA endothelium and platelets can be mediated by RAGE receptors [114]. Their ligands and advanced glycation end products (AGEs), include HMGB1, Mac-1, phosphatidylserines, and lipopolysaccharides (LPS) [114,115]. PA endothelium contains at least two RAGE isoforms, and expression of these increases when stimulated with pro-inflammatory cytokines and erythrocytes during blood transfusion, resulting in the expression of adhesion molecules on the surface [114,115].

HMGB1 via interaction with RAGE activates platelets and makes them stimulate neutrophils to release NETs [116]. NETs exert prothrombotic effects by promoting blood coagulation, adhesion and aggregation of platelets (mainly due to histones H3 and H4) and recruiting red blood cells [116,117]. Blood coagulation is supported by NETs ability to compartmentalize thrombus-associated tissue factor pathway inhibitor (TFPI) and facilitate its degradation by neutrophil-derived serine proteases [118]. NET components, including histones, myeloperoxidase, neutrophil elastase, and cathepsin G, cause a cytotoxic effect and contribute to pulmonary epithelium and endothelium damage [51]. Their immediate involvement in thrombosis has been shown both in DVT and in thrombosis of the coronary arteries [116,117]. NETs have been identified in organized thrombi in the PA, where they are located extra- and intracellularly [97]. Evidence of NETs with fibrin fibers and the involvement of neutrophils and CD4 monocytes has also been reported in patients with COVID-19 with thrombosis in the minor branches of the PA [77].

It has been shown that histones circulating in the blood after injuries, sepsis, and other abnormal conditions can become a source of damage to the pulmonary epithelium and endothelium [119]. After severe injury, histone concentration in blood increases in 4 h, reaches its peak by 24 h, and still remains high 72 h later [119]. High concentrations of histones become toxic to the PA endothelium, and in one third of patients with toxic histone concentrations, lung injuries are diagnosed [119,120]. Histones rapidly bind to endothelium but can be blocked by preincubation with an anti-histone antibody [119]. Prolonged incubation with histones causes endothelial death [121]. Administration of sublethal doses of histones to mice is accompanied by accumulation of neutrophils in alveolar micro-vessels, vacuolization of the pulmonary epithelium and endothelium, intra-alveolar hemorrhages, and formation of thrombi rich in fibrin and platelets [120].

## 5. The Role of Microparticles in the Development of PA Thrombosis

Microparticles (MPs) can play an important role in thrombotic pulmonary disease. Their sources are platelets, endotheliocytes, and many other cells [51]. Microparticles retain the lipid bilayer of the parent cells and may contain their RNA, enzymes, and mediators [51]. In healthy people, circulating MPs predominantly originate from platelets; a lesser number is derived from endothelial cells [122,123]. Endothelial cells activated by MPs secrete pro-inflammatory cytokines, including IL-1, IL-6, IL-8, and Monocyte Chemoattractant Protein 1 (MCP-1) [122]. Due to the high concentration of the TF and vWf on the surface of some MPs, they can induce thrombin generation [122,124,125]. TF-positive endothelial microparticles have adhesion molecules on their surface, which recruit monocytes and platelets, activate them, and transfer TF to their surface [126,127]. Three-fold higher blood concentrations of platelet-derived and TF-positive MPs are observed in patients with thrombosis of the PA branches rather than that in healthy individuals. This is accompanied by a 5-fold increase of chances for thrombosis [128,129]. The concentration of platelet MPs and TF-positive MPs remains fatally high during the first 3 months after VTE and later on it significantly drops [129].

## 6. Conclusions

**Figure 2:**
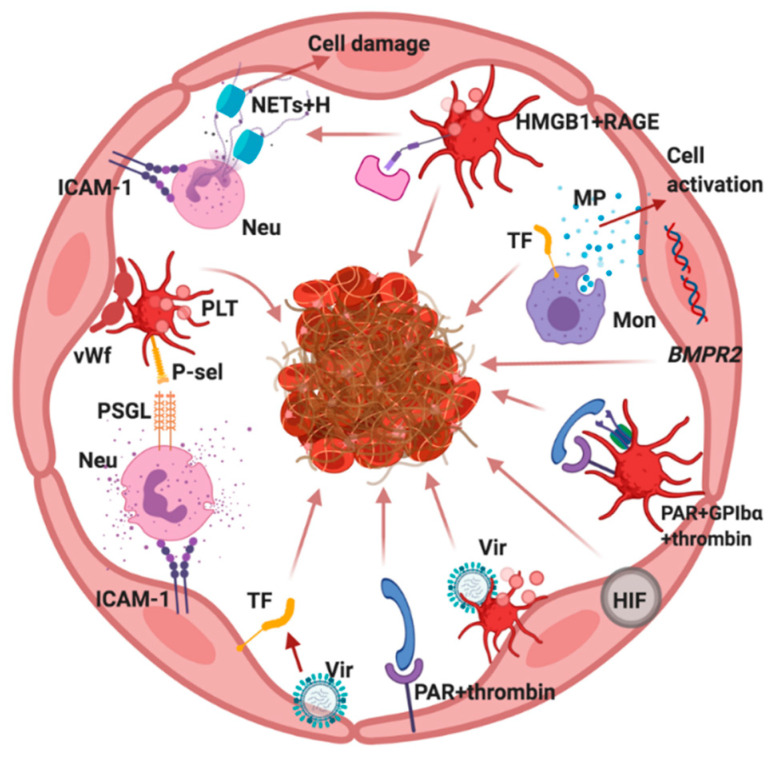
Possible mechanisms of pulmonary artery (PA) thrombosis.

Both clinical and experimental data indicate that different mechanisms can result in thrombus formation in the PA (Figure 2). Histological evaluations of its thrombosed branches confirm the existing signs of endothelitis with edema, desquamation, apoptosis of endotheliocytes, and infiltration of the vascular wall with leukocytes, lymphocytes, and megakaryocytes. During activation, the endothelium loses its anticoagulant properties and acquires a procoagulant phenotype, which precedes thrombus formation. Thus, a condition that could be designated as thrombotic angiopathy may underlie local thrombosis in the PA. 

The most frequent initiating factor for PA thrombosis is an infectious agent or a systemic inflammatory process. Hemodynamic factors may be less important for the initiation of PA thrombosis, but they do affect permeability of the vascular wall and can lead to the progression of thrombosis. 

Recruitment of platelets, endothelium, and leukocytes, upregulation of the proinflammatory cytokine levels, and secretion of adhesion molecules, which collectively trigger the coagulation cascade and induce thrombosis, suggest that the term “immunothrombosis” is applicable to PA occlusion in situ. 

Further studies are required to establish differential diagnostic criteria and peculiarities of the pathogenesis of the two conditions, PA thrombosis and PA thromboembolism, and this may become a basis for development of separate therapeutic approaches to treat these diseases.

NETs + H,NETs (neutrophil extracellular traps and histones); HMGB1+RAGE, interaction of HMGB1 and RAGE receptors; neutrophil (Neu); microparticles (MPs); tissue factor (TF); platelet (PLT); von Willebrand factor (vWf); P-selectin (P-sel); P-selectin glycoprotein ligand (PSGL); interaction of neutrophil with ICAM-1; monocyte (Mon); Bone Morphogenetic Protein Receptor Type 2 (BMPR2); PAR + platelet glycoprotein Ibα (GPIbα) + thrombin, interaction of thrombin with PAR and GPIbα; virus (Vir); hypoxia inducible factor (HIF); PAR + thrombin, interaction of thrombin with PAR.

## Abbreviations

VTE	Venous thromboembolism
DVT	Deep vein thrombosis
PA	Pulmonary artery
PE	Pulmonary embolism
COPD	chronic obstructive pulmonary disease
CT	Computed tomographic imaging
SCA	Sickle cell anemia
APC	Activated protein C
EPCR	Endothelial Protein C Receptor
eNOS	Endothelial nitric oxide synthase
MP	Microparticles
PAR	Protease mediated receptor
NO	nitric oxide
TNFα	tumor necrosis factor
ROS	reactive oxygen species
TF	tissue factor
ICAM	Inter-Cellular Adhesion Molecule
VCAM	Vascular cell adhesion molecule
tPA	Tissue plasminogen activator
uPA	urokinase-type
HIFs	hypoxia inducible factors
NF-kB	nuclear factor-KappaB
TFPI	Tissue factor pathway inhibitor
SMC	smooth muscle cells
VEGF	Vascular endothelial growth factor
ACEII	angiotensin converting enzyme
PSGL-1	P-selectin glycoprotein ligand
vWf	von Willebrand factor
HMGB1	high-mobility group box 1
RAGE	receptor for advanced glycation end products
NETs	neutrophil extracellular traps
TLR	Toll-like receptor
CD40L	CD40 ligand
IFITM3	Interferon-induced transmembrane protein 3
CLR	C-type lectin receptor
Mac-1	Macrophage-1 antigen
MCP-1	Monocyte Chemoattractant Protein 1
BMPR2	Bone Morphogenetic Protein Receptor Type 2
